# Targeting RNA-Binding proteins Roquin-1 and Regnase-1 could enhance CAR-iPSC-derived macrophage immunotherapy for solid tumors: a perspective and challenges

**DOI:** 10.1080/15476286.2025.2581385

**Published:** 2025-10-28

**Authors:** Fatemeh Mirzaei, Andisheh Mosaffa Jahromi, Haniyeh Molavi, Dieter Kabelitz, Kurosh Kalantar, Seppo Meri

**Affiliations:** a Department of Immunology, School of Medicine, Shiraz University of Medical Sciences, Shiraz, Iran; b Institute of Immunology, Christian-Albrechts University of Kiel and University Hospital Schleswig, Kiel, Germany; c Department of Bacteriology & Immunology and the Translational Immunology Research Program (TRIMM), University of Helsinki, Helsinki, Finland; d Autoimmune Diseases Research Center, Shiraz University of Medical Sciences, Shiraz, Iran

**Keywords:** Solid tumours, CAR-macrophages (CAR-iMacs), Roquin-1, Regnase-1, immunotherapy

## Abstract

Solid tumours present major treatment obstacles because of their immunosuppressive microenvironment and poor response to traditional chimeric antigen receptor (CAR)-based immunotherapies. Recent advances in cellular engineering have introduced CAR-macrophages derived from induced pluripotent stem cells (CAR-iMacs) as a promising approach to get around these obstacles. CAR-iMacs are designed to attack tumours, but their phenotypic plasticity can cause them to transform into M2-like macrophages in the tumour environment (TME), where they may instead suppress immune responses and promote tumour progression and metastasis. Roquin-1 and Regnase-1 are RNA-binding proteins that act as negative regulators of inflammatory genes that contribute to the phenotypic plasticity of macrophages. This perspective highlights a novel approach to augmenting anti-tumour responses of CAR-iMacs by simultaneously knocking out Roquin-1 and Regnase-1 via CRISPR-Cas9 gene editing. This approach drives a shift from an immunosuppressive M2-like state to an M1 state, promoting sustained pro-inflammatory signalling, boosting phagocytic and cytotoxic capabilities within the tumour microenvironment. Addressing a serious constraint in conventional adoptive cell therapies, this dual-targeting platform could provide a potent and scalable immunotherapeutic treatment for solid malignancies.

## Introduction

Cancer is responsible for approximately 10 million annual deaths globally. Of these, solid malignancies comprise nearly 85%, emphasizing the critical need for novel approaches in therapeutic intervention [1]. While traditional cancer treatments, including recently established CAR therapies, have significantly progressed, their success in treating solid tumours remains limited [2].

Solid tumours exhibit a hostile tumour microenvironment (TME) characterized by a dense extracellular matrix that impedes immune cell infiltration and drug delivery, heterogeneous antigen expression enabling immune escape, and immunosuppressive elements (e.g. regulatory T cells, myeloid-derived suppressor cells (MDSCs), and M2-polarized macrophages) that secrete inhibitory cytokines [3]. Metabolic constraints in TME, like hypoxia, further impair immune function, while chronic antigen exposure drives exhaustion via upregulation of inhibitory receptors [4]. Tumour plasticity also fosters rapid therapy resistance, and the TME often limits the persistence of adoptively transferred cells, including those delivered in CAR therapies [5]. To overcome these barriers, CAR-engineered macrophages (CAR-M) especially iPSC-derived CAR-Macrophages (CAR-iMac) emerge as a promising new opportunity for cancer immunotherapy [6]. Recent advances in iPSC technology and CRISPR-Cas9 gene editing enable precise engineering of these cells [7]. For instance, knockout of immunosuppressive genes such as Roquin-1 and Regnase-1 can shift macrophage polarization from the pro-tumour M2 phenotype to the tumoricidal M1 state [8,9]. This dual strategy combining CAR targeting with gene editing aims to remodel the TME and enhance anti-tumour immunity, addressing the core limitations of current immunotherapies.

## Car therapies: from CAR-T cells to CAR-iMac

CAR is a genetically engineered receptor that enables immune cells to specifically recognize tumour-associated antigens independently of MHC presentation, endowing CAR-T cells with potent tumour killing capacity [10]. CAR-T cell therapies have demonstrated significant efficacy in haematologic malignancies, while their effectiveness against solid tumours is constrained by challenges such as impaired immune cell trafficking, limited infiltration into the TME, and the immunosuppressive nature of the TME [11]. To overcome the limitations of CAR T-cell therapies in solid tumours, CAR macrophages are emerging as a promising cellular immunotherapy for solid tumours, offering a unique functional profile distinct from CAR-T cells. CAR-M represent a promising therapeutic approach to overcome key limitations of lymphoid-based CAR therapies in solid tumours. CAR-M exhibit distinct functional advantages, including potent phagocytic activity, enhanced antigen presentation, and the ability to infiltrate and remodel the TME. These properties underscore their potential as a viable strategy for solid tumour immunotherapy [6]. However, despite their therapeutic promise, CAR-M therapies encounter several challenges, including limited scalability, donor-dependent variability in primary cell sourcing, and difficulties in sustaining a pro-inflammatory M1 phenotype within the immunosuppressive TME [12,13]. To address these limitations, induced pluripotent stem cell (iPSC)-derived CAR macrophages (CAR-iMacs) have emerged as a scalable, standardized, and customizable alternative [12]. Similarly, to other genetically engineered CAR-expressing cells, the activity of CAR-iMacs is defined by the specificity of the engineered receptor. When CAR-iMacs directed against tumour-associated antigens that are selectively or highly expressed on malignant cells, their pro-inflammatory and phagocytic functions remain largely restricted to the tumour microenvironment [14]. However, if the target antigen is also present on normal tissues, off-tumour effects may occur [15]. Careful antigen selection and advanced CAR designs, including dual-targeting or inhibitory modules, are therefore critical to improve safety and therapeutic precision [6].

The persistence of CAR-iPSC-derived macrophages (CAR-iMacs) in the tumour microenvironment has been observed to last at least 30 days, enabling sustained antitumor effects [14,16]. However, long-term persistence and its potential to cause chronic inflammation remain under investigation. Prolonged presence of M1-polarized macrophages may increase the risk of inflammatory side effects, suggesting that additional treatments or controlled dosing regimens could be required to balance efficacy and safety. Notably, CAR-iMacs are expected to contract after tumour clearance, potentially minimizing long-term inflammatory risks as seen in related adoptive cell therapies. Therefore, careful monitoring and treatment optimization are crucial for clinical translation [17,18].

Nevertheless, CAR-iMacs still face obstacles, such as functional exhaustion, cytokine-mediated suppression, and insufficient persistence of inflammatory signalling in the hostile TME [19].

A critical concern is the phenotypic plasticity of macrophages, wherein a high infiltration of CAR-iMacs into tumours may lead to their differentiation into immunosuppressive M2-like macrophages, paradoxically promoting tumour progression and metastasis. Thus, developing strategies to sustain the pro-inflammatory M1 phenotype of CAR-iMacs is imperative to maximizing their efficacy as a next-generation cellular therapy for solid tumours [20].

## Targeting Roquin-1 and Regnase-1: a promising strategy to maintain the inflammatory phenotype of CAR-iMacs

Roquin-1 (Rc3h1) and Regnase-1 (also known as Zc3h12a or MCPIP1) are RNA-binding proteins which critically regulate overlapping sets of inflammatory mRNAs through recognition of stem-loop (SL) structures in the 3’ untranslated regions (UTRs) of target mRNAs [21]. In macrophages Roquin-1 regulates inflammatory mRNAs, contributing to immune homoeostasis. Its disruption can enhance inflammatory gene expression, affecting macrophage polarization and function [22,23]. Regnase-1 also targets mRNAs encoding cytokines such as IL-6, TNF-α, IL-12 and other inflammatory mediators, preventing excessive inflammation. For instance, Regnase-1 knockout in macrophages leads to hyperinflammation and autoimmunity due to prolonged cytokine mRNA stability. The role of Regnase-1 and Roquin-1 in suppressing pro-inflammatory cytokine production suggests that they may limit M1 polarization in CAR-iMacs, which could favour an M2-like, immunosuppressive phenotype in the TME. Furthermore, maintaining a pro-inflammatory, anti-tumour M1-like phenotype in CAR macrophages is critical for their therapeutic efficacy [24]. Research on CAR-T cells also shows that disrupting Regnase-1 and Roquin-1 boosts T-cell inflammatory responses and antitumor activity, suggesting that targeting these RNA-binding proteins in CAR-iMacs could be a promising approach [25]. Also, in engineered T cells targeting tumour antigens, knocking out both Roquin-1 and Regnase-1 led to a significant increase in T cell accumulation within tumours and reduced expression of exhaustion markers, resulting in improved tumour clearance [26]. Targeting this regulatory axis in CAR-iMacs might enhance their functional polarization towards an M1 phenotype, augment phagocytic capacity, and improve persistence within the immunosuppressive TME. Supporting this approach, a recent study demonstrated that Regnase-1 knockdown promotes a shift towards M1 polarization by suppressing NF-κB signalling, while concurrently upregulating C/EBPβ and PPARγ pathways [9]. Moreover, knockout or functional inhibition of Roquin-1 and Regnase-1 could prolong the expression of M1-associated transcripts such as IL-6, TNF-α, and iNOS, while maintaining the surface expression of costimulatory molecules such as CD80 and CD86 [27,28]. This would enhance the cytotoxic and phagocytic functions of CAR macrophages within the tumour microenvironment. Furthermore, sustaining an M1 phenotype could improve antigen presentation and promote a pro-inflammatory milieu that supports adaptive immune responses.

While targeting Roquin-1 and Regnase-1 May enhance the persistence of a tumoricidal M1 phenotype, it could also raise safety concerns. Given their role as post-transcriptional repressors of inflammatory genes, deletion of these proteins leads to hyperinflammation and autoimmune phenotypes in preclinical models [9,26,29]. Prolonged stabilization of cytokine transcripts such as TNF-α, IL-6, and IL-12 could theoretically lead to systemic inflammatory responses or collateral tissue damage. Nevertheless, these risks may be mitigated in CAR-iMacs, since activation is largely restricted to tumour-associated antigens and localized by the tumour microenvironment. Moreover, safety switches such as inducible suicide genes could be engineered into CAR-iMacs to provide an additional safeguard against uncontrolled inflammation.

## Proposed methodology: targeting Roquin-1 and Regnase-1 in CAR-iMac using CRISPR-Cas9 to overcome immunosuppression in solid tumour microenvironment

**Figure 1.**
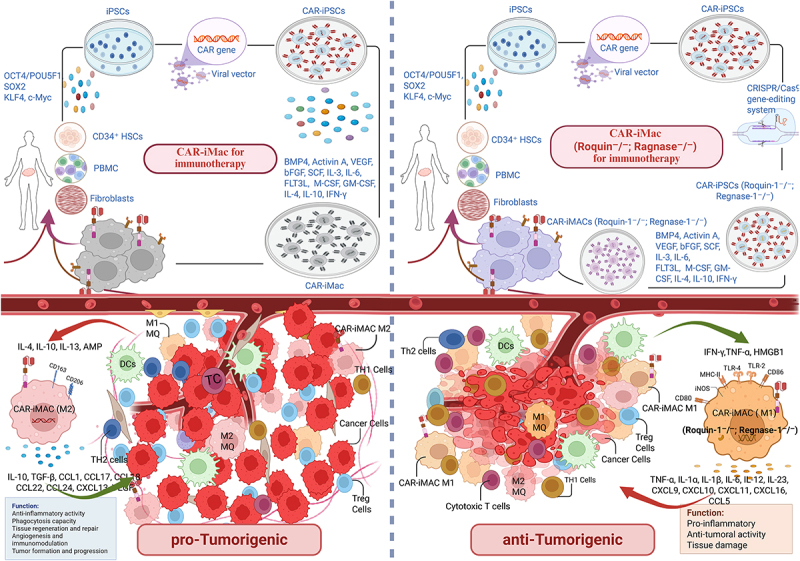
Proposed strategy to overcome the phenotypic skewing of CAR-iMacs towards an M2-like state in immunosuppressive tme, by targeting Roquin-1 and Regnase-1 using CRISPR-Cas9. CAR-iMacs have overcome many of the limitations faced by CAR-T and CAR-Mɸ cells; however, the immunosuppressive tumour microenvironment in solid tumours may drive their plasticity towards an M2-like phenotype. It can paradoxically promote tumour formation and progression. To enhance the anti-tumour efficacy of CAR-iMacs against solid tumours, we propose a gene-editing strategy in which Roquin-1 and Regnase-1 are knocked out using the CRISPR-Cas9 system. This approach can permanently disrupt specific genes, ensuring that macrophages differentiate into a persistent, highly pro-inflammatory M1 state, which strongly correlates with anti-tumour activity. The figure was created with BioRender software.

To enhance the anti-tumour efficacy of CAR-iMacs against solid tumours, a gene editing strategy is proposed in which Roquin-1 and Regnase-1 are knocked out (Figure 1). Three primary gene-editing techniques exist for altering Roquin-1 and Regnase-1 in CAR-iMacs [7,30,31]. First, small molecules enable reversible and dynamic control of macrophage polarization pathways. However, the off-target effects and systemic toxicity restrict the clinical application of small molecular inhibitors [32]. Second, RNA interference (RNAi) technology provides transient gene silencing with rapid deployment. This approach is accompanied by risks of incomplete efficacy and off-target transcript suppression. It also requires repeated dosing, which limits its application [33]. Third, and possibly the most thrilling aspect, is the CRISPR-Cas9 system, which can permanently knock out specific genes in a heritable manner, effectively securing differentiation of macrophages into a persistent, elevated pro-inflammatory M1 state that strongly correlates with an anti-tumour function [34]. Despite ongoing worries regarding off-target impacts and genomic instability, recent progress in techniques such as advancements in high-fidelity base/prime editors and improved delivery systems (e.g. AAVs or LNPs) has minimized genomic instability risks [35]. The ability of CRISPR for multiplexed editing also enables the simultaneous targeting of Roquin-1 and Regnase-1, providing precision and scalability to create CAR iMacs that could withstand tumour-mediated reprogramming with vital benefit for immunotherapy in solid tumours.

To initiate the process, iPSCs would be generated from somatic cell sources, including peripheral blood mononuclear cells (PBMCs) or CD34^+^ haematopoietic stem cells isolated from umbilical cord blood [36,37]. These iPSCs would be genetically engineered to express a CAR specific for a tumour-associated antigen of choice, for instance mesothelin, which is relevant in ovarian cancer. Following CAR integration, CRISPR-Cas9-mediated gene disruption will be performed to target the RNA-binding or RNase domains of Roquin-1 and Regnase-1, thereby preventing degradation of pro-inflammatory cytokine mRNAs and promoting stabilization of M1-associated transcripts. To minimize off-target effects, high-specificity single-guide RNAs (sgRNAs) could be designed to selectively target Roquin-1 and Regnase-1. For genome editing, CRISPR-Cas9 ribonucleoprotein complexes (RNPs) will be delivered into CAR-expressing iPSCs via optimized electroporation or lipid nanoparticle-mediated transfection [38]. Following CRISPR-Cas9-mediated gene disruption, a homogeneous population of double knockout cells can be obtained through an enrichment strategy. Edited cells co-expressing a fluorescent or selectable marker are typically isolated by fluorescence-activated cell sorting (FACS) or antibiotic selection [39,40]. The double knockout status of Roquin-1 and Regnase-1 can be confirmed by sequencing and protein-level validation (e.g. Western blot or qPCR) [41]. This enrichment step is important to eliminate partially edited cells that may retain residual Roquin/Regnase activity and display undesired M2-like polarization tendencies [42]. By gene editing, CAR-iPSCs are subsequently differentiated into M1-polarized CAR-expressing macrophages (CAR-iMacs-M1) through a 20–30-day protocol utilizing defined cytokine cocktails (GM-CSF + IFN-γ) [38]. The resulting population typically displays high purity ( > 90% CD11b^+^ CD14^+^) and characteristic M1 markers such as iNOS and HLA-DR, accompanied by robust secretion of pro-inflammatory cytokines (TNF-α, IL-12p70) as measured by ELISA. Stable CAR expression can be verified by flow cytometry and qPCR [43]. Functional validation generally involves quantification of phagocytic activity using pHrodo-labelled tumour target cells in co-culture assays and live-cell imaging to assess tumour cell clearance. Cytokine profiling via multiplex analysis confirms maintenance of the M1 phenotype, characterized by elevated TNF-α and IL-12 with low IL-10 levels. Transcriptomic analyses further support M1 polarization through increased expression of IRF5 and STAT1 [14].

To evaluate cytotoxic activity, three-dimensional tumour spheroid models or tumour tissue organoids could be employed, with lactate dehydrogenase (LDH) release or DNA staining assays used as quantitative readouts of tumour cell killing [44]. For *in vivo* validation, orthotopic mouse models of solid tumours can be employed to evaluate tumour infiltration, immune activation, and tumour regression [14]. Recruitment of endogenous immune cells to the tumour microenvironment could also be monitored. This approach enables a relatively comprehensive functional and therapeutic assessment of Roquin-1- and Regnase-1-deficient CAR-iMacs-M1 with the aim to elucidate their potential as a robust platform for solid tumour immunotherapy.

## Conclusion

To summarize, in our view the engineering of CAR macrophages derived from iPSCs, utilizing CRISPR-Cas9 to knockout Roquin-1 and Regnase-1, presents a viable approach to overcome the immunosuppressive tumour microenvironment. This method promotes consistent M1 polarization, boosts anti-tumour response, and mitigates major drawbacks seen in existing cell therapies. The targeted manipulation of these two genes is especially crucial, allowing macrophages to preserve their tumour-killing characteristics amid solid tumours. The precise targeting of these two genes is particularly significant, as it enables macrophages to maintain their tumoricidal phenotype within solid tumours, offering a novel and potentially transformative solution for solid tumour immunotherapy.

## Data Availability

Data sharing not applicable to this article as no datasets were generated or analysed during the current study.
